# Socio-behavioural factors and early childhood caries: a cross-sectional study of preschool children in central Trinidad

**DOI:** 10.1186/1472-6831-13-30

**Published:** 2013-07-09

**Authors:** Rahul Naidu, June Nunn, Alan Kelly

**Affiliations:** 1 Faculty of Medical Sciences, The University of the West Indies, St. Augustine, Trinidad and Tobago; 2 School of Dental Sciences, Trinity College Dublin, Dublin 2, Ireland; 3 Department of Public Health & Primary Care, Trinity College Dublin, Dublin 2, Ireland

**Keywords:** Early childhood caries, Preschool children, Oral health behaviour, West Indies

## Abstract

**Background:**

Early childhood caries (ECC) is a public health problem due to its impact on children’s health, development and well being. Little is known about early childhood oral health in the West Indies or the influence of social and behavioural factors on the prevalence and severity of early childhood caries in this preschool population. The aims of this study were to describe the prevalence and severity of ECC in preschool children in a region of central Trinidad and to explore its relationship with social and behavioural factors.

**Method:**

A cross-sectional survey was undertaken on children aged 3-5 years-old from a random sample of preschools in central Trinidad. Oral health examinations were conducted for children for whom parental consent was given, using WHO criteria (visual diagnosis / cavitation at d3). A self-reported questionnaire was distributed to all parents and caregivers. Variables included socio-demographics, oral health knowledge, attitudes and behaviours, visible caries experience and treatment need.

**Results:**

251 children were examined, 50.2% were male with a mean age of 3.7 years (SD 0.67) and 71% were of Indian ethnicity. The prevalence of ECC was 29.1% and the prevalence of severe early childhood caries (S-ECC) was 17.5%. 29.9% of children had some treatment need, with 12% in need of urgent care or referral. Poisson generalized linear mixed model analysis found a higher rate of visible caries experience for children who ate sweet snacks more than twice a day (p < 0.001), had poorer parental dental health ratings (p < 0.0001), a previous dental visit (p < 0.0001) and difficulty finding dental care (p < 0.001).

**Conclusion:**

The prevalence and severity of ECC in central Trinidad was related to oral health behaviours and access to dental care. Oral health promotion should include more supportive and practical advice for parents and caregivers of preschool children along with improved access to dental care to enable primary prevention and management of ECC.

## Background

Early childhood caries (ECC) has been defined as ‘the presence of one or more decayed, missing due to caries, or filled tooth surfaces in any primary teeth in children under 6 years of age’ [1,2]. In its more rampant form it is now described as severe early childhood caries (S-ECC), which is defined for 3–5 year-olds as one or more cavitated, (missing due to caries) or filled smooth surface in primary maxillary anterior teeth or a decayed, missing or filled tooth score of ≥ 4 (age 3), ≥ 5, (age 4), or ≥ 6 (age 5) [2]. This replaces older terms such as ‘nursing-bottle caries’ and ‘baby-bottle-tooth decay’. There is still some variation due to differences in case-definitions. Internationally, the prevalence of ECC has been reported to range from 6-90%, with most developed countries in the lower end, and most developing countries, in the middle to higher end of this range [3]. Within-country disparities are also common, with preschool children from disadvantaged communities generally experiencing higher levels of disease than the general population [4,5].

Due to its high prevalence, impact on quality of life, potential for increasing risk of caries in the permanent dentition and role in oral health inequalities, ECC is recognised as a serious public health problem [5]. Socio-economic, socio-cultural and socio-behavioural determinants are believed to influence specific risk factors for ECC such as dietary and feeding practices, oral hygiene and dental attendance patterns [6,7]. These risk factors do not work independently but are likely to have a complex interplay. Fisher-Owens et al. [8] describe a multilevel conceptual model to explain influences at the child, family and community level. At the child level, risk factors include: genetic, biological, social and physical environment and health behaviours. At the family level, they include: socioeconomic status, family function and health behaviours, and at the community level: culture, social capital, fluoridation and the healthcare system.

### ECC in the West Indies and children’s oral health in Trinidad and Tobago

There is a paucity of data on ECC in the Caribbean region. In Anguilla, caries prevalence among children aged 36–71 months was 21% with S-ECC affecting 17% of the sample [9].

Trinidad and Tobago is a twin-island, English-speaking democratic republic in the West Indies. At the last census the total population was 1.3 million with 25% under the age of 15 [10]. Arising from its colonial history, the country has a multi-ethnic composition, with people of Indian, African and mixed descent being the main ethnic groups. Approximately one quarter of the population live in rural areas [10].

There are currently no published epidemiological data on the oral health of preschool children in Trinidad and Tobago. However, the persistence of untreated caries in the primary dentition of school-aged children can be considered a public health problem, as a national survey in Trinidad reported almost two thirds of 6–8 year-olds had caries experience [11]. Acute problems arising from decayed primary teeth were also the most frequent cause of emergency dental visits in a dental hospital clinic [12]. Risk models have shown that decayed teeth presenting at primary school-age indicate these children are likely to have been at high risk for caries during their pre-school years [13]. Furthermore, a preliminary Trinidadian-based study, among a sample of parents and caregivers attending a dental hospital clinic, exposed confusion, lack of accurate information and low awareness of preventive dental care for preschool children [14].

Determining the role of social and behavioural risk factors on oral disease levels and outcomes can help inform oral health policy and in particular, the development of appropriate oral health promotion strategies.

### Aim

The aims of this study were to describe the prevalence and severity of ECC among preschool children in Trinidad and explore the relationship between ECC and social and behavioural determinants.

## Methods

A cross-sectional oral health survey of preschool children was undertaken in the Caroni region of central Trinidad. The Caroni region can be considered one of the more populous regions of the island, and home to a mixture of urban and rural communities with a broad socioeconomic spectrum. The accessible population were children aged 3-5-years, attending preschools in the Caroni Education District, registered with the Trinidad and Tobago Ministry of Education. Preschools in the Caroni region are generally situated in urban centres however, children attending these preschools are drawn from across the education district catchment area, which includes children living in rural home addresses. Based on this list, there were 27 government/government-assisted and 57 non-government preschools in the District at the time of the survey, with an enrolled population of approximately 2000 children.

### Sample selection

A required sample size of 250 was based on an estimated caries prevalence of 30% (using data from neighbouring islands) and a 6% level of precision. Assuming an average of 30 children per preschool and approximately 20% non–response rate, this required 10 preschools in the sample. Very small schools (enrolment <15) and very large schools (enrolment >60) were excluded from the sampling frame. This was done to include preschools of similar sizes in the sample and facilitate data collection by a single examiner. Drawn from the remaining list of preschools in the sampling frame, the final random selection resulted in a mix of 3 government/government-assisted and 7 non-government preschools.

### Permission and liaison

Ethical approval for the study was obtained from the University of the West Indies Faculty of Medical Sciences, Research Ethics Committee. Permission was sought from the head teachers / administrators of each selected preschool, for their preschool to take part in the study. For preschools that agreed, individual parents and caregivers were invited by letter to complete a self-administered questionnaire and provide written positive consent for an oral examination of their child / children.

### Instruments and variables

The self-administered parent/caregiver questionnaire was based on a previous instrument used in Trinidad [14]. Children were also assessed for behaviour rating using the Frankl Behaviour Rating Scale at the point of attempting oral examination [15].

The following variables were included.

Questionnaire variables: Parent/ caregiver age, sex, ethnicity, occupation of head of household, level of education, rating of child’s dental health, oral health knowledge beliefs and practices, child age, ethnicity, sex, health and development.

Clinical variables: Dentition status and treatment need and need for urgent care or referral, based on WHO 1997 criteria [16]. Specific codes from the dentition status were subsequently used for calculation of the ‘dmft’ index score, to indicate caries experience. For the (d) component, this included dentition status: ‘Decayed’ and ‘Filled with decay’, for the (f) component: ‘Filled, no decay’ and for (m) component: ‘Missing as a result of caries’. Primary teeth considered to have been lost due to trauma were coded separately and not included in the count for caries experience.

Treatment need was assessed for each tooth immediately after the tooth status was recorded. Treatment need categories included: no-treatment, preventive care, one-surface restoration, two or more-surface restoration, crown, pulp care, extraction. A child was recorded as in need of urgent care / referral for dental treatment if it was assessed that pain, infection or serious illness might have otherwise occurred in a short period of time (few days to a month).

### Examination protocol

Oral examinations were undertaken by a single trained and calibrated examiner (RN) working with an assistant/data recorder. Dental caries was measured using WHO 1997 criteria but without the use of an explorer [16]. Caries was recorded at the d3 level (cavitation into dentine). Teeth were not dried but soft debris on tooth surfaces was removed with a cotton roll or gauze square and assessed visually, using a disposable mouth mirror. Children were examined in their preschool classroom using natural light, in a seated position on a small chair/bench with the examiner positioned behind. A second attempt was made to examine children who initially refused (this second attempt followed completion of oral examinations on compliant children whom the previously non-compliant children were able to observe). Before the examinations a dental health presentation was given in each preschool using puppets. After the oral examinations, all children were given colouring books and a toothbrush.

The questionnaire and clinical protocol were piloted in a single preschool not included in the final sample. Minor changes were made to the questionnaire following the pilot phase to improve question clarity. Data were processed and analysed using SPSS version 16 and R statistical program version 2.13.1.

### Statistical analysis

Means, standard deviations and 95% confidence intervals were calculated for continuous variables and proportions with 95% confidence intervals for categorical variables. Pearson Chi-square was employed in the bivariate analyses comparing proportions with visible caries experience and no visible caries experience. Statistical significance was set at p<0.05.

In the multiple variate analyses, visible caries experience (dmft) was modelled both as a Poisson generalized linear mixed model (GLMM) for actual counts of dmft and a Logistic GLMM for presence and absence of visible caries experience. Children were considered nested within preschool clusters to control for similarities between children attending the same preschools. The analysis was conducted using the package lme4 in R [17]. Potential model predictors were recoded as required by collapsing sparsely populated levels. To achieve a parsimonious model, only model terms with confirmed significance at an alpha of 5% were retained in the final model based on changes in the Akaike Information Criterion [18]. However, for comparative purposes, the Logistic model results retained the same set of variables as for the Poisson model. Missing values were assumed to be missing completely at random and so were excluded from the modelling.

The Kappa statistic was used to assess intra-examiner reliability.

## Results

### Response to the survey

Nine preschools took part in the study. From an enrolment of 340 children, 314 parents gave consent for the oral examination (92% parental response rate). Of these children, 36 (11.5%) were absent on the day and 27 (8.6%) refused examination, being recorded as ‘definitely negative’ behaviour’ on the Frankl Behaviour rating scale. Two hundred and fifty one children completed the oral examination having shown ‘positive’ or ‘definitely positive’ behaviour.

### Demographics of children who completed the oral examination

The age range of the 251 children who completed oral examinations was 3 to 5 years with a mean age of 3.7 years (SD 0.67). One hundred and twenty six (50.2%) were male. Seventy per cent were of Indian ethnicity, 18% mixed and 9.6% of African ethnicity. Health or development concerns were reported in 14% of children, with issues regarding general health the most commonly reported concern (8.4%) followed by problems with hearing and eyesight (as reported by parents/caregivers).

### Examiner reliability

Examiner reliability was tested by re-examination of children at one preschool (25 children). Re-examinations took place on the same day as data collection, after an interval, having examined all the children once. The Kappa statistic for intra-examiner reliability (caries experience) was 0.9.

### Visible caries experience (ECC)

The frequency distribution of caries experience was highly positively skewed with most children (70.9%) having no visible caries experience (dmft = 0). Seventy three children (29.1%) children had some visible caries experience (dmft > 0), with similar proportions among male and female children (29.4% and 28.8%, respectively). The mean dmft for the whole sample was 1.40 (SD 3.01, 95% CI: 1.03-1.78). When children with no visible caries experience (dmft = 0) were excluded, the mean dmft for the remainder of the sample (dmft > 0) was 4.82 (SD 3.83, 95% CI: 3.93-5.72) with the majority of this being untreated decay (dt) mean 4.71 (SD 3.80, 95% CI: 3.82-5.60).

### Severe early child hood caries (S-ECC)

Forty four children (17.5%) had evidence of severe early childhood caries i.e. one or more cavitated, missing due to caries, or filled primary maxillary anterior teeth. This definition was adapted from the AAPD/AAP definition [2] to account for use of whole tooth score rather than surface score.

### Treatment need

Seventy five children (29.9%) had some treatment need beyond routine preventive care. The majority of this need (88%) was for one or two surface restorations and 36% for pulp care or extraction. Children may have required more than one treatment type so the percentages do not total to 100. Thirty children (40% of those with some treatment need), were in need of urgent care, mostly due to caries and its sequelae (pulp infection and abscess).

### Social and behavioural factors

#### Bivariate model

The null hypothesis used in these analyses was that there were no social and behavioural differences between the proportions of children with visible caries experience and those without visible caries experience. In the bivariate analyses no significant associations were found for proportions of children with/without visible caries experience and socio-demographic variables, socioeconomic status, oral health knowledge, oral hygiene and fluoride use, breastfeeding history and use of a feeding bottle.

Table 1 describes significant associations between the proportion of children with visible caries experience and socio-behavioural factors. The proportion of children with visible caries experience was significantly associated with parents who rated their child’s oral health as fair to poor, the child having visited a dentist or dental nurse, those who had difficulty in finding dental care and children with more frequent intake of fruits and sweet snacks.

**Table 1:** Socio-behavioural factors and proportions of children with visible caries N = 251

**Oral health knowledge, attitudes and dental attendance**	**No visible caries experience (dmft = 0)** **n (%)**	**Visible caries experience (dmft > 0)** **n (%)**	**p-value (Chi square)**
**How would you rate your child’s dental health**			
Excellent	41 (23.3)	7 (9.7)	
Very good	63 (35.5)	15 (20.8)	
Good	59 (33.5)	20 (27.8)	
Fair	12 (6.8)	19 (26.4)	
Poor	1 (0.6)	11 (15.3)	<0.001
**Has your child ever visited a dentist or dental nurse**			
Yes	50 (28.7)	37 (51.4)	
No	124 (71.3)	35 (48.6)	<0.01
**Have you ever had difficulty in finding dental care for your child**			
Yes	14 (8.5)	14 (20.3)	
No	151 (71.3)	55 (79.7)	0.02
**How often does your child have fruits**			
Never	2 (1.1)	0 (0)	
Rarely	11 (6.2)	5 (6.8)	
Once a day	93 (52.5)	22 (30.1)	
Twice a day	41 (23.2)	21 (28.8)	
More than twice a day	30 (16.9)	25 (34.2)	
Don’t know	0	0	<0.01
**How often does your child eat sweet snacks**			
Never	2 (1.1)	1 (1.4)	
Rarely	48 (27.1)	10 (14.1)	
Once a day	80 (45.2)	26 (36.6)	
Twice a day	29 (16.4)	11 (15.3)	
More than twice a day	18 (10.1)	23 (32.4)	
Don’t know	0	1 (1.4)	<0.01

#### Multiple variate models

The Poisson generalized linear mixed model of dmft counts (Table 2) shows the rate of visible caries experience (dmft) nearly doubles for children who have 2 or more sweet snacks daily as compared to those who never or rarely eat sweet snacks. As general dental health rating improves, the rate of visible caries experience drops significantly for dental health rating: average, good or excellent, compared to: fair or poor. Those children with no previous experience of dental care had only half the rate of visible caries experience compared to those with previous experience of dental care. The rate of visible caries experience also reduced considerably for children who had no difficulty in finding dental care as compared to those who did.

**Table 2:** Estimated rate of visible caries experience based on Poisson generalized linear model with children nested in preschools

**Term**	**Estimated rate of visible caries experience (dmft)**	**95% Confidence nterval**	**p-value**
Intercept	2.58	0.95 - 7.00	0.06
Frequency sweet snacks =1 daily (base = rarely or never)	0.98	0.67 - 1.41	0.90
Frequency sweet snacks = 2 > daily (base = rarely or never)	1.85	1.32 - 2.58	<0.001
Dental health rating by parent = average (base = fair or poor)	0.12	0.16 - 0.30	<0.0001
Dental health rating by parent = good (base = fair or poor)	0.22	0.08 - 0.17	<0.0001
Dental health rating by parent = excellent (base = fair or poor)	0.17	0.11 - 0.27	<0.0001
Previous dental visit = no (base = yes)	0.49	0.38 - 0.64	<0.0001
Difficulty finding dental care = no (base = yes)	0.65	0.48 - 0.86	<0.001

In the Logistic Poisson generalized linear mixed model for presence/absence of visible caries experience (Table 3), frequency of sweet snacks and difficulty in finding dental care were not significant. The remaining terms (dental health rating and previous dental experience) remained significant with results in good agreement with the Poisson model.

**Table 3:** Estimated odds ratio for presence versus absence of visible caries experience based on a Logistic generalized linear mixed effects model with children nested in preschools

**Term**	**Estimated odds ratio**	**95% Confidence interval**	**p-value**
Intercept	1.40	0.11 – 17.16	0.792
Frequency sweet snacks =1 daily (base = rarely or never)	1.10	0.45 – 2.68	0.840
Frequency sweet snacks = 2 > daily (base = rarely or never)	2.13	0.86 – 5.25	0.102
Dental health rating by parent = average (base = fair or poor)	0.20	0.08 – 0.50	0.001
Dental health rating by parent = good (base = fair or poor)	0.12	0.05 – 0.30	<0.001
Dental health rating by parent = excellent (base = fair or poor)	0.11	0.03 – 0.34	<0.001
Previous dental visit = no (base = yes)	0.13	0.17 – 0.68	0.002
Difficulty finding dental care = no (base = yes)	0.47	0.19 – 1.20	0.115

## Discussion

The response rate for the oral examinations was affected mainly by absence of children on the day of the visit, followed by non-compliance. Although designed for assessment of child behaviour during dental treatment, the Frankl scale [15] was used in this study to assess compliance with the oral examination. Non-compliance (Frankl score ‘definitely negative’) was considered an indication of Dental Behaviour Management Problems (DBMP). DBMP in preschool children can have implications for service provision with respect to choice of treatment, need for specialist referral and additional clinical resources. A measure of DBMP should therefore form part of the oral health assessment for young children [19].

Comparison of the present data with international caries data for preschool children must take account of methodological differences such as use of national survey data versus sub-samples, differing age ranges, ECC definitions and examination criteria. Despite these considerations, some overall trends are apparent. The prevalence of ECC in the present study was similar to a neighbouring English-speaking Caribbean island [9] but much lower than that seen in some other developing countries around the world [20,21]. Interestingly, the prevalence of caries experience in Trinidad was also similar to recent data reported in the UK [22]. Almost a fifth of children in the present study showed evidence of S-ECC, again similar to previously-reported Caribbean data [9].

Consistent with most of this ECC experience attributable to decayed teeth (dt), the majority of children who were in need of treatment in the present study required restorative care, with a smaller proportion needing pulp-care or extraction. Importantly, over a third of children with unmet treatment need required urgent care, to address problems resulting from untreated caries such as dental abscess.

Debate has arisen over the most effective and appropriate methods for addressing untreated caries in the primary dentition of young children. Some research has shown that the majority of un-restored carious primary teeth remain symptomless until exfoliation [23]. However, the carious primary teeth most likely to go on to cause pain, are molars affected soon after eruption, which progress rapidly to large cavities [24]. In addition, the risk of young children experiencing pain and sepsis increases with higher caries experience, suggesting that children at high risk for ECC would benefit the most from dental intervention [25].

Data from the last national oral health survey of schoolchildren in Trinidad and Tobago indicated that the majority of treatment need was for restorations and extractions in the primary teeth of 6–8 year-olds [11]. This again suggests that much of the ECC that develops in the preschool period remains untreated into the early school years. This situation can lead to a ‘downward spiral’ of delayed care-seeking for ECC and symptom-based attendance for dental care [25], as evidenced by ECC related problems being the most frequent reason for attendance at emergency departments [26]. Based on the analysis in the present study, both having difficulty in finding dental care and having attended a dentist were related to a higher rate of visible caries experience. This finding may suggest problems with access to dental care as well as some children attending due to a caries-related problem, as symptoms such as pain or infection would be more likely in children with a higher rate of caries experience. This would be consistent with the issue of symptom-based dental visits for ECC [25,26].

Prevention and management of ECC should therefore involve early dental attendance, that is, by 12 months of age, to establish a ‘dental home’ early in a child’s life. The ‘dental home’ can enable ‘anticipatory guidance’ caries risk assessment and early intervention for dental problems [27-29].

Although there is a well-established link between lower SES and higher caries experience in young children [30] this was not a finding of the present study. This may have been due to the sample size and/or lack of sensitivity with respect to the proxy variables, ‘highest level of education’ and ‘parent/ caregiver occupation’. Use of household-income may have been more discriminating but collection of this information was considered too intrusive. However, consistent with the literature on dietary practices was the finding of a significantly greater proportion of children with visible caries experience among those who ate sweet snacks ‘twice’ or ‘more than twice’ a day, Greater frequency of sweet snacks was also associated with a higher rate of visible caries experience in the Poisson regression model.

Lower oral health ratings were associated with a higher rate of caries experience in the Poisson regression model. This generally accurate parental perception of the child’s oral health status is consistent with the literature, as parental oral health ratings of their children have been shown to correlate with actual clinical status and need for dental treatment [31].

In a recent systematic review it was concluded that parental oral health behaviour was an intermediary in the development of ECC, being the result of their oral health knowledge, attitudes and beliefs, which in turn are influenced by education, socioeconomic status and culture [32]. This was found to be consistent with the findings of the present study where parent/caregiver oral behaviours such as dental attendance and dietary habits were associated with a higher rate of visible caries experience in the Poisson regression model. To be effective, oral health promotion should therefore include an understanding of parent and caregiver knowledge and attitudes [33] and oral health advice must be clear and appropriate [34].

Adopting a more client-centred approach, which considers underlying social determinants of health, may help to motivate parents and caregivers to adopt healthier dental health behaviours for their families. One such approach is motivational interviewing (MI), a counselling method that attempts to elicit behaviour change by resolving ambivalence and increasing self-efficacy [35].

### Limitations of the study

The possibility of selection bias must be considered in this study. Not all children in this age group attend preschools or day-care facilities and their socio-demographic / behavioural characteristics may have differed from those children sampled through preschool enrolment. Also, caries experience and socio-behavioural factors among children who refused or were not present on the day for the oral examination (almost of fifth of those for whom parental consent was given), may have differed from those who were examined. Bias may also have arisen from exclusion of very small and very large preschools from the sampling frame. Furthermore, although possibly representative of preschool children in the Caroni education district of central Trinidad, these data may not reflect the oral health of all preschool children across the nation. Associations between caries experience and socio-behavioural factors may have differed if non-cavitated lesions were included.

Being cross-sectional in design the context and temporal effect of the identified risk factors is not clear. This may have also resulted in apparent paradoxes in the final regression model which included theoretical antecedent events (frequency of sweet snacks), concurrent events (parental assessment of child oral health) and downstream events (dental attendance).

## Conclusions

The prevalence and severity of ECC in this sample of preschool children in Trinidad suggest the need for improved access to dental care and more effective oral health promotion for this population group. This should include primary prevention and interventions that offer more practical advice and support for parents and caregivers, including counselling type approaches that consider the social determinants of oral health.

## Abbreviations

AAPD: American Academy of Paediatric Dentistry; AAP: American Academy of Paediatrics; DBMP: Dental behaviour management problems; Dmft: decayed, missing and filled teeth; ECC: Early childhood caries; S-ECC: severe early childhood caries; WHO: World Health Organisation.

## Competing interests

The authors declare that they have no competing interests.

## Authors' contributions

RN conducted the fieldwork and data collection. RN and JN contributed to the design of the study, RN, JN and AK contributed to data analysis / interpretation and preparation of the manuscript. All authors read and approved the manuscript.

## Pre-publication history

The pre-publication history for this paper can be accessed here:

http://www.biomedcentral.com/1472-6831/13/30/prepub
